# Audio-Visual Causality and Stimulus Reliability Affect Audio-Visual Synchrony Perception

**DOI:** 10.3389/fpsyg.2021.629996

**Published:** 2021-02-18

**Authors:** Shao Li, Qi Ding, Yichen Yuan, Zhenzhu Yue

**Affiliations:** Department of Psychology, Sun Yat-sen University, Guangzhou, China

**Keywords:** stimulus reliability, complex stimuli, audio-visual synchrony, audio-visual causality, audio-visual integration

## Abstract

People can discriminate the synchrony between audio-visual scenes. However, the sensitivity of audio-visual synchrony perception can be affected by many factors. Using a simultaneity judgment task, the present study investigated whether the synchrony perception of complex audio-visual stimuli was affected by audio-visual causality and stimulus reliability. In Experiment 1, the results showed that audio-visual causality could increase one's sensitivity to audio-visual onset asynchrony (AVOA) of both action stimuli and speech stimuli. Moreover, participants were more tolerant of AVOA of speech stimuli than that of action stimuli in the high causality condition, whereas no significant difference between these two kinds of stimuli was found in the low causality condition. In Experiment 2, the speech stimuli were manipulated with either high or low stimulus reliability. The results revealed a significant interaction between audio-visual causality and stimulus reliability. Under the low causality condition, the percentage of “synchronous” responses of audio-visual intact stimuli was significantly higher than that of visual_intact/auditory_blurred stimuli and audio-visual blurred stimuli. In contrast, no significant difference among all levels of stimulus reliability was observed under the high causality condition. Our study supported the synergistic effect of top-down processing and bottom-up processing in audio-visual synchrony perception.

## Introduction

Most events in daily life come from multiple sensory modalities, and people often need to integrate the information of different sensory channels to form a consistent and unified representation in time. Such an experience makes people mistakenly believe that multi-sensory stimuli in events seem to be synchronous, but this is not necessarily the case. It is almost impossible for audio-visual stimuli from the same event to reach the corresponding sensory pathways at the same time due to the difference between the speed of light and the speed of sound in physical propagation, as well as the speed of neural processing and conduction. The time interval between onsets of visual stimuli and auditory stimuli is called audio-visual onset asynchrony (AVOA). People can automatically integrate asynchronous audio-visual signals to form an audio-visual synchrony perception if a visual stimulus and an auditory stimulus are presented in a certain temporal window (Poeppel, 2005; Vatakis and Spence, 2006; Keetels and Vroomen, 2012; Vatakis, 2013). Based on previous studies, the temporal window of integration (TWI) could be calculated according to the point of subjective simultaneity (PSS) and the just noticeable difference (JND), that is, TWI = [PSS − JND, PSS + JND] (Kostaki and Vatakis, 2018; Paraskevoudi and Vatakis, 2019). Different JNDs of audio-visual cross-modal integration have been reported in previous studies (Vroomen and Keetels, 2010). For example, Hirsh and Sherrick (1961) adopted a temporal order judgment (TOJ) task to investigate cross-modal integration. They found that the JNDs of participants were approximately 20 ms for audio-visual stimuli. In contrast, by using similar simple stimuli such as noise bursts and light flashes, Keetels and Vroomen (2005) investigated how spatial disparity influenced audio-visual temporal judgment. When visual stimuli and auditory stimuli originated from the same location, they reported JNDs of about 50 ms.

Previous studies have revealed that synchrony perception could be affected by bottom-up factors, e.g., the modality of stimuli, stimulus type, stimulus intensity, duration of stimuli, and so on (Lewald and Guski, 2003; Stevenson and Wallace, 2013; Chan et al., 2014; Eg and Behne, 2015). For example, when a visual stimulus precedes an auditory stimulus, the TWI of participants is wider than *vice versa* (Lewald and Guski, 2003; Vatakis et al., 2008). In addition, the width of the TWI is also affected by the stimulus intensity. For low-intensity (e.g., dark) stimuli, the TWI is wider than that for high-intensity (e.g., bright) stimuli (Fister et al., 2016). In addition, compared with non-speech stimuli, people are more tolerant of AVOA of speech stimuli, manifesting in a wider TWI (Dixon and Spitz, 1980; Stevenson and Wallace, 2013). For example, by adopting both the simultaneity judgment (SJ) task and TOJ task, Stevenson and Wallace (2013) investigated TWIs of different stimulus types (simple flash beeps, dynamic handheld tools, and single syllable utterances). They found that the width of the TWI was not significantly different between non-speech stimuli of flash beeps and tools, whereas the width of TWI of speech stimuli (syllable utterances) was significantly larger than that of non-speech stimuli.

In addition, stimulus reliability, i.e., the clarity or recognizability of stimuli, has been found to affect synchrony perception, and inconsistent results have been found for audio-visual synchrony perception. For example, the speech information received by people with visual or auditory impairment may be unreliable. To understand information from unreliable stimuli, one of the strategies adopted by the brain is to use visual cues (i.e., lip reading) to facilitate auditory comprehension (Bernstein et al., 2004; Ma et al., 2009). Participants can predict auditory stimuli accurately in the presence of more visual cues. Some studies have found that people's sensitivity to AVOA of visually blurred stimuli is smaller than that of visually intact stimuli (Magnotti et al., 2013; Eg et al., 2015) because blurred stimuli cannot provide enough visual cues for participants. That is, a decrease in stimulus reliability makes participants more tolerant of AVOA. However, other researchers found the opposite results (Shahin et al., 2017; Shatzer et al., 2018). Shatzer et al. (2018) manipulated stimulus reliability by using blurred visual stimuli and distorted auditory stimuli. Participants were required to perform a speech SJ task, and the authors found that participants were more tolerant of AVOA for non-blurred stimuli. These inconsistent results might be due to the stimulus vagueness adopted in the previous studies. Stein and Stanford (2008) found that multisensory integration can affect the neurons' responses, which relies on the relative physiological salience. When the visual or auditory cues are weak, the neural responses of multisensory neurons involved in the integration is “superadditive.” That is, the gain of multisensory integration is higher than the sum responses elicited by uni-sensory stimuli.

Audio-visual synchrony perception could also be biased by top-down factors, e.g., prior experience and audio-visual causality (Levitin, 2000). For example, musician experts are more sensitive to synchrony perception than average people (Petrini et al., 2009). Recent studies have shown that audio-visual causality, that is, the degree of causality between visual and auditory stimuli, significantly affects audio-visual synchrony perception. By adopting a novel experimental paradigm, Levitin (2000) found the effect of audio-visual causality on synchrony perception of action stimuli. In this experiment, two participants acted either as an actor or an observer and wore headphones to receive auditory sounds. Action executors waved a hammer to hit the desktop, and the observer watched alongside. When the hammer hit the table, two people could hear synchronous or several different levels of asynchronous sound. Participants were required to judge whether the sound heard from headphones and action hitting the table were synchronous. The actor of the action understood the causal relationship between the action and the sound better than the observer; thereby, the actor was more accurate and more sensitive to synchrony perception than the observer. Similar findings were also found regarding speech stimuli, in which the integration of audio-visual information could improve perceptual accuracy when only one person was speaking (Ma et al., 2009). In contrast, when there are two speakers—that is, the causal relationship between the speaker and the sound is relatively unclear—the integration of audio-visual information can reduce perceptual accuracy (Shams and Beierholm, 2010).

It is plausible that people are more likely to detect asynchronization between visual and auditory stimuli when the causal relationship between them is clear. However, none of the studies mentioned above manipulated audio-visual causality directly. In a high causality condition, the observer could form an expectation according to the visual or auditory information, which could be used to suitably judge whether the stimuli from two modalities were synchronized. Therefore, in the present study, by manipulating audio-visual causality directly, we investigated how audio-visual causality impacts audio-visual synchrony perception.

The first aim of the present study is to investigate the effect of audio-visual causality on synchrony perception. In the present study, audio-visual causality was manipulated by the relationship between visual and auditory stimuli in the experiment. Moreover, we are also interested in the co-effect of audio-visual causality and stimulus reliability on synchrony perception. In Experiment 1, visual and auditory stimuli were presented, and participants were required to perform a simultaneity judgment (SJ) task. Two kinds of visual or auditory stimuli were used: action and speech stimuli. According to the findings of previous studies (Levitin, 2000; Eg and Behne, 2015), the higher the predictability is, the more sensitive participants would be to AVOA. We hypothesized that audio-visual causality affected the sensitivity of synchrony perception. That is, under a low causality condition, people would be more tolerant of AVOA and would be more likely to experience synchrony perception than they would in a high causality condition. In Experiment 2, by adopting an SJ task of speech, we investigated the interaction between stimulus reliability and audio-visual causality with blurred visual or auditory stimuli. If the reliability of visual or auditory stimuli was weakened, it would be easy for people to make simultaneity judgments, i.e., reducing the sensitivity to AVOA. Thus, we hypothesized that stimulus reliability had a significant influence on the sensitivity of the synchronized perception of speech as a bottom-up factor. That is, the lower the stimulus reliability is, the more tolerant people would be to AVOA, and the more likely they would be to make synchronous judgments. Moreover, an interaction effect between stimulus reliability and audio-visual causality is expected. Under a low causality condition, participants more easily make synchronous judgments with less stimulus reliability, whereas under a high causality condition, stimulus reliability does not affect synchrony perception.

## Experiment 1

The aim of Experiment 1 is to explore the influence of audio-visual causality on synchrony perception. Moreover, according to previous studies (Levitin, 2000; Petrini et al., 2009), audio-visual synchrony may vary as a function of stimulus type. Thus, to explore the role of audio-visual causality in different types of stimuli, both action and speech stimuli were adopted (Vatakis and Spence, 2006; Ma et al., 2009; Eg and Behne, 2015). Two levels of causality were used. Using “knocking on a door” for example, in a high causality scene, a hand would be seen knocking on a door and a clear tap-tap sound would be heard. Participants could understand and judge whether the visual stimulus was synchronized with the auditory stimulus (Vroomen and Stekelenburg, 2010). By contrast, in the condition of low causality, the hand of the actor would be seen turning the doorknob, and no knocking action would be seen. In this situation, participants could not predict the sound accurately.

### Methods

#### Participants

Referring to the effect size obtained from previous studies (Eg and Behne, 2015), we estimated that the effect size was 0.5, and the power was 0.8. Through GPower calculation, the sample size required for Experiment 1 was determined to be more than 26. Twenty-nine participants (5 males, Mean_age_ = 19.97 years old, SD = 1.56 years old) completed the experiment. Data from two participants were deleted because they were beyond three standard deviations, and 27 participants were included in the analysis. All the participants were native Chinese speakers with normal or corrected-to-normal vision and normal hearing. They signed informed consent forms before participating in the experiment. They received 50 RMB after completing the experiment. The study was conducted according to the guidelines in the Declaration of Helsinki (2013) and was approved by the Ethics Committee of the Department of Psychology, Sun Yat-sen University.

#### Materials

Four kinds of homemade audio-visual clips were involved: high causality/action, high causality/speech, low causality/action and low causality/speech. The content of twelve audio-visual clips was shown in the whole experiment (see Supplementary Material). The vocabulary of speech stimuli was selected from the Chinese lexical database compiled by Sun et al. (2018). To control the familiarity of these materials, we conducted Mann-Whitney U tests and found no significant difference between the word frequency of the two kinds of speech stimuli (*p* = 0. 513) (see Supplementary Material). For the action stimuli, the collision of objects and the sound produced by objects were clearly shown in the high causality audio-visual clips, whereas the sound of the internal object motion or indirect action was included in the low causality clips. For the speech stimuli, the initials of every word in the audio-visual clips of high causality are bilabial sounds with distinct mouth shapes (e.g., b, p, m), which makes it easy for people to discriminate sounds according to the movement of the mouth, whereas sounds with no obvious movement of lips (e.g., z, s, d, t) were used in the low causality clips.

All original audio-visual clips were recorded in a bright and quiet room with a Huawei Mate 20 mobile phone. The person performing the actions and reading the words in the clips was the same young male. Stimulus onset asynchronies (SOAs) were 0 ms for the original clips. Adobe Premiere Pro 2020 was used to generate another four audio-visual clips of different SOAs for each segment: −400, −200, 200, and 400 ms (the “–” means that the auditory stimulus precedes the visual stimulus). Each audio-visual clip was uniformly processed so that hands or mouth movement could be presented at the center of the picture, with a length of 3 s. The video resolution was 900 × 900 pixels, the frame rate was 30 FPS and the audio sampling rate was 48 kHz (dual-channel). Then, MATLAB 2017b was used to equalize the sound volume of all audio-visual clips to the same level. Finally, a total of 60 audio-visual clips were obtained as stimuli for Experiment 1.

#### Procedure and Experimental Design

Two within-participants variables were used as independent variables: Audio-visual causality (high and low) and Stimulus type (action and speech). The dependent variables were point of subjective simultaneity (PSS) and just noticeable difference (JND). PSS measures the degree of asynchrony for each individual's perception of time consistency. The closer the PSS is to 0, the closer the person's perception of simultaneity is to objective reality. JND measures the perception sensitivity of participants.

**Figure 1:**
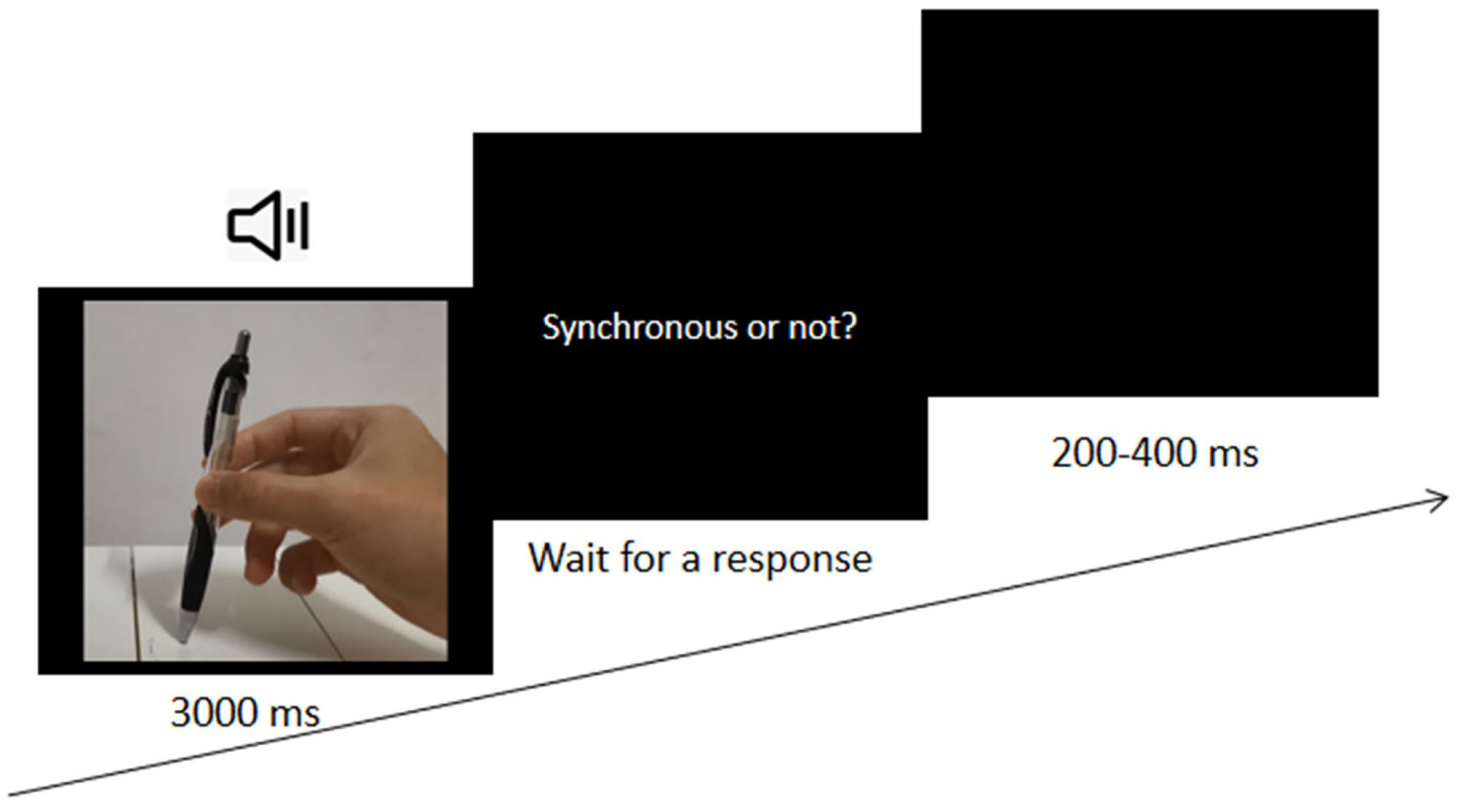
The procedure of Experiment 1.

An SJ task was used in the present study. Five SOAs (−400, −200, 0, 200, and 400 ms) between auditory stimuli and visual stimuli were used. Participants were to practice first for 24 trials, and only those with a response accuracy rate >75% could begin the formal experiment. Otherwise, they needed to practice again, and those who failed to pass 3 times could not perform the formal experiment. The formal experiment consisted of five blocks, with 120 trials in each block. All trials were presented in a pseudorandom sequence. In each trial, an audio-visual clip of 3 s was presented, and participants needed to determine whether the visual stimulus and auditory stimulus were synchronous. They were required to press “Y” if they determined that the auditory and visual actions or speech stimuli were synchronous or “N” for asynchronous perception. Then, a black screen was displayed for 200–400 ms before the next trial (see Figure 1). At the end of each block, participants could rest.

#### Data Analysis

**Figure 2:**
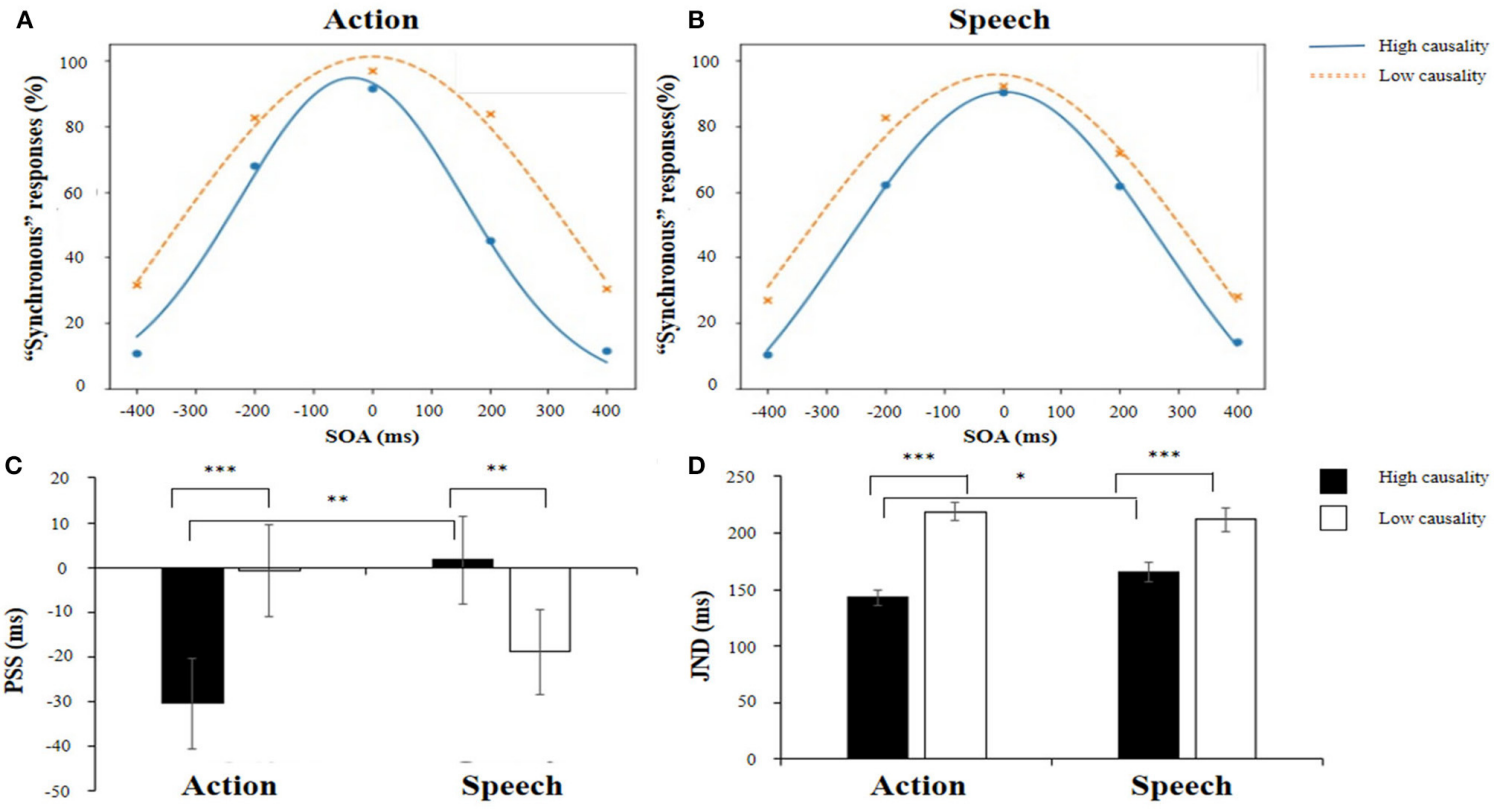
Scatter plots and Gaussian fitting curves of the percentage of synchronous responses for action stimuli **(A)** and speech stimuli **(B)**. **(C)** Point of subjective simultaneity (PSS) under each experimental condition. “-” indicates that auditory stimuli were presented ahead of visual stimuli. **(D)** Just noticeable difference (JND) at each experimental condition. *p < 0.05, **p < 0.01, and ***p < 0.001.

We calculated the average percentage of “synchronous” responses of each experimental condition (see Figure 2). To obtain JND and PSS data, a scatter plot was calculated by taking SOA as the X axis and the percentage of synchronous responses as the Y axis. Then, Gaussian curve fitting was conducted, which has usually been used in previous studies (Vatakis et al., 2008; Eg and Behne, 2015). The PSS was the peak of the Gaussian curve, at which subjects most likely perceived audio-visual stimuli as synchronous. JNDs were calculated by subtracting the PSS from the X value corresponding to a Y value of 75%, reflecting the sensitivity of the subjects' perception of audio-visual simultaneity (Vroomen and Keetels, 2010).

Thus, we conducted 2 × 2 repeated measures ANOVA on PSS and JND values using Jamovi 1.1.9, with two within-participants variables: Audio-visual causality (high and low) and Stimulus type (action and speech). Multiple comparisons are based on Tukey's T test.

### Results

The mean PSS and JND under each experimental condition are shown in Table 1. The fitted Gaussian curves of each experimental condition are shown in Figure 2. We used the coefficient of determination (*r*^2^) to assess the goodness of fit of the Gaussian curves. The mean *r*^2^ was 0.99 for Action_high causality and Speech_high causality and 0.97 for Action_low causality and Speech_low causality.

**Table 1:** The mean value and standard error of the point of subjective simultaneity (PSS) and just noticeable difference (JND) under each experimental condition.

		**PSS**		**JND**	
**Audio-visual causality**	**Stimulus type**	**Mean**	**SE**	**Mean**	**SE**
High causality	Action	−30	10.09	143	6.68
	Speech	2	9.78	165	8.52
Low causality	Action	−1	10.30	219	7.79
	Speech	−19	9.47	212	10.60

For PSSs, a 2 (Audio-visual causality: high/low) × 2 (Stimulus type: action/speech) repeated-measures ANOVA revealed a significant interaction between Audio-visual causality and Stimulus type [*F*_(1,26)_ = 39.99, *p* < 0.001, $\eta_{p}^{2}$ = 0.606]. Further analysis showed that the PSS of the action stimulus was significantly different from that of the speech stimulus in the high causality condition, *t* = −3.79, *p* = 0.003*, d* = 0.73 (see Figure 2C). In contrast, in the low causality condition, the PSS of the action stimulus did not differ from that of the speech stimulus. Moreover, for action stimuli, the PSS in the high causality condition (M = −30 ms) was significantly different from that in the low causality condition (M = −1 ms), *t* = −4.82, *p* < 0.001*, d* = 0.93, indicating that participants perceived audio-visual stimuli as synchronous when audition preceded vision. However, for speech stimuli, the PSS with high causality (M = 2 ms) was significantly different from the PSS with low causality (M = −19 ms), *t* = 3.35, *p* = 0.008*, d* = 0.64. In addition, the main effects of Audio-visual causality [*F*_(1,26)_ = 0.93, *p* = 0.344, $\eta_{p}^{2}$ = 0.034] and Stimulus type were not significant [*F*_(1,26)_ = 0.88, *p* = 0.358, $\eta_{p}^{2}$ = 0.033].

For the JND, the 2 (Audio-visual causality: high and low) × 2 (Stimulus type: action and speech) repeated-measures ANOVA revealed that the main effect of Audio-visual causality was significant [*F*_(1,26)_ = 91.11, *p* < 0.001, $\eta_{p}^{2}$ = 0.778], indicating that the JND with high causality (154 ms) was significantly smaller than the JND with low causality (215 ms). Moreover, the interaction between Audio-visual causality and Stimulus type was significant [*F*_(1,26)_ = 10.39, *p* = 0.003, $\eta_{p}^{2}$ = 0.285]. Further analyses showed that the JND of action stimuli was significantly smaller than that of speech stimuli in the high causality condition (see Figure 2D), *t* = −2.81, *p* = 0.035*, d* = 0.54. However, in the low causality condition, the JNDs between action stimuli and speech stimuli were not significant. Moreover, for the action stimuli, the JND in the high causality (143 ms) condition was significantly smaller than that in the low causality (219 ms) condition, *t* = −9.67, *p* < 0.001, *d* = 1.86. For the speech stimuli, the JND in the high causality (165 ms) condition was also significantly smaller than the JND in the low causality (212 ms) condition, *t* = −5.99, *p* < 0.001, *d* = 1.15. However, the differences in JNDs between the two causality conditions for speech stimuli were significantly smaller than those for action stimuli. In addition, the main effect of Stimulus type was not significant [*F*_(1,26)_ = 1.39, *p* = 0.248, $\eta_{p}^{2}$ = 0.051].

### Discussion

The results in Experiment 1 showed that audio-visual synchrony perception was affected by audio-visual causality. Moreover, such an effect was modulated by the stimulus type. For the JND, participants' JNDs were larger in the low causality condition than in the high causality condition for both action and speech stimuli, indicating that participants were more sensitive to synchrony perception in the high causality condition. Our results are consistent with the findings of Levitin (2000), in which the observer's JND is larger than that of the actor who used hammer to hit the table because the actor better understands the causal relationship between the action and the sound. Similarly, Vatakis et al. (2012) found that for speech stimuli, the participants' JND was smaller when they observed visually salient bilabial syllables than velar and alveolar syllables, which were less visible. Moreover, our results show that in the high causality condition, the mean JNDs of action stimuli are smaller than those of speech stimuli. During the perception of action, accurate perception of AVOA is more important than that for speech perception. In addition, previous studies show that complex language requires a wider TWI for phonological classification (Virginie, 2013). Insensitive synchrony may be useful in the understanding of speech and more tolerant of AVOA. In other words, longer AVOA in language perception may be more evolutionarily adaptive.

For PSS, participants' synchrony perception of action stimuli was close to the objective simultaneity in the low causality condition, whereas participants' synchrony perception of action stimuli was less accurate in the high causality condition. These results indicate that audio-visual causality can bias synchrony perception. For action stimuli, we observed a negative PSS in the high causality condition but not in the low causality condition. That is, participants considered a video with sound preceding the visual stimulus as a subjectively coincident stimulus, which could be partly due to the high predictivity between visual stimuli and auditory sound in the high causality condition. In contrast, for the speech stimuli, participants' synchrony perception was close to the objective simultaneity in the high causality condition, whereas participants' synchrony perception of action stimuli was less accurate in the low causality condition. Vatakis and Spence (2006) found that the PSS under each experimental condition was between −80 and 70 ms. Similarly, in the study of Eg and Behne (2015), the PSS was between −90 and 240 ms (also see Dixon and Spitz, 1980). Thus, the small PSS in the present study could be attributed to the control of audio-visual causality, which makes participants' simultaneity judgment close to reality. In addition, participants could use visual cues, such as lip reading, to promote auditory understanding (Bernstein et al., 2004; Ma et al., 2009). In the present study, obvious lip movement was involved in the high causality condition, whereas inconspicuous lip movement was involved in the low causality condition. Therefore, participants could not predict the synchrony of auditory and visual stimuli in the low causality condition due to insufficient visual cues.

## Experiment 2

Although people are less sensitive to AVOA of speech stimuli in Experiment 1, the difference between speech and non-speech stimuli may have been confounded by the complexity of stimuli (Vroomen and Stekelenburg, 2011). In their study, the authors compared the PSS and JND between the participants who marked sine wave speech (SWS) as a language and the participants who marked SWS as an artificial computer sound and found no difference in synchrony perception between the two participant groups.

The aim of Experiment 2 is to further explore the influence of audio-visual causality on synchrony perception by adopting speech stimuli. In addition, stimulus reliability was also manipulated to investigate the co-effect of top-down and bottom-up factors during audio-visual synchrony perception. For speech stimuli, audio-visual causality was controlled by the visual cues in the speech. Some phonemes have more obvious lip shapes and are easier to recognize, i.e., higher causality, such as /b/, /m/, etc. (Cappelletta and Harte, 2012). In contrast, for low causality speech stimuli, fewer visual cues were provided, and it is difficult to predict auditory stimuli according to visual stimuli, such as /d/, /t/, etc. Thus, we hypothesized that stimulus reliability had less of an effect on synchrony perception when more cues were provided by the high causality stimulus.

### Methods

#### Participants

Referring to the effect size obtained from previous studies (Shahin et al., 2017; Shatzer et al., 2018), we estimated that the effect size was 0.6, and the power was 0.8. Through GPower calculation, the sample size required for experiment 2 was more than 24. Thus, thirty participants (11 males, Mean_age_ = 20.13 years old, SD = 1.18 years old). All participants were native Chinese speakers with normal or corrected-to-normal vision and normal hearing. They signed informed consent forms before participating in the experiment. They received 50 RMB after completing the experiment. The study was conducted according to the guidelines in the Declaration of Helsinki (2013) and was approved by the Ethics Committee of the Department of Psychology, Sun Yat-sen University.

#### Materials

Twelve homemade audio-visual clips of speech stimuli were involved in Experiment 2, either high causality or low causality (see Supplementary Material). Vocabulary in speech stimuli was selected from the Chinese lexical database compiled by Sun et al. (2018). We also controlled the familiarity of these materials as in Experiment 1 (*p* = 0.631) (see Supplementary Table 1 for the word frequency). The causality of speech was manipulated as in Experiment 1.

The original audio-visual clips were the same as in Experiment 1. Visual stimuli were presented before auditory stimuli; thus, three SOAs were adopted as in Shatzer et al. (2018). The SOA was 0 ms for the original clips. Adobe Premiere Pro 2020 was used to generate another two audio-visual clips of different SOAs for each segment (200 and 400 ms). The clear original video was blurred by MATLAB 2017B with Gaussian blur processing (filter size = 65 × 65 pixels, standard deviation = 15 pixels). The clear original sound was sampled down to 2 kHz to obtain a blurred sound, and the volume of all sound was homogenized to the same level. Then, Ffmpeg 4.2.2 was used to combine the visual stimuli and auditory stimuli into 4 audio-visual segments with different stimulus reliability levels: audio-visual intact, visual_intact/auditory_blurred, visual_blurred/auditory_intact, and audio-visual blurred. Finally, a total of 144 audio-visual clips were obtained as materials for Experiment 2.

#### Procedure and Experimental Design

Three within-participant variables were adopted as independent variables: SOA (0, 200, and 400 ms), Audio-visual causality (high and low) and Stimulus reliability (audio-visual intact, visual_intact/auditory_blurred, visual_blurred/auditory_intact, and audio-visual blurred). The dependent variable was the percentage of “synchronous” responses.

**Figure 3:**
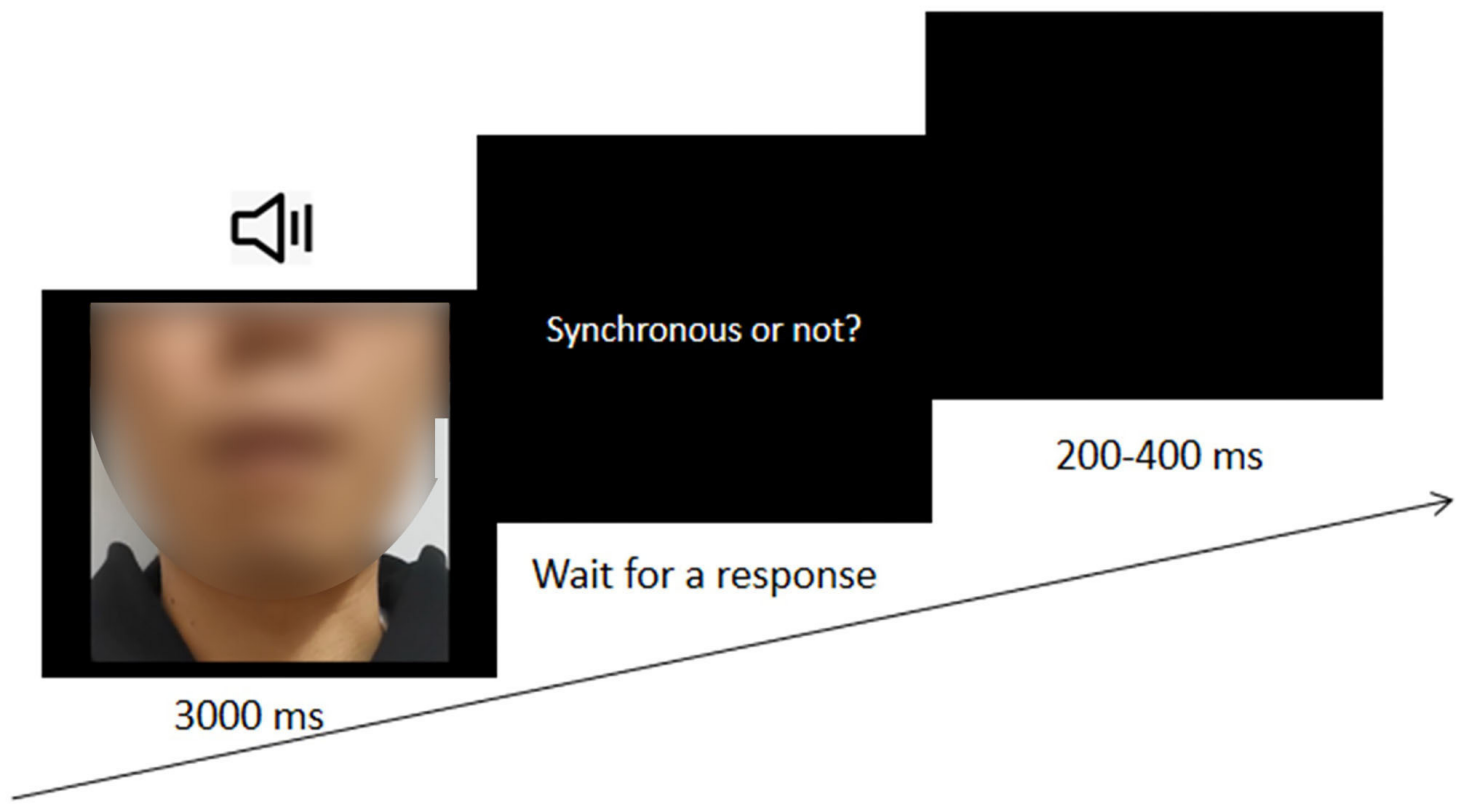
The procedure of Experiment 2.

The formal experiment consisted of six blocks, with 144 trials in each block. All trials were presented in a pseudorandom sequence. In each trial, an audio-visual clip of 3 s was presented first, and participants were required to judge whether the visual stimulus and auditory stimulus were synchronous (see Figure 3). Participants practiced before the formal experiment, and other settings were the same as in Experiment 1.

#### Data Analysis

The mean percentage of “synchronous” responses of each experimental condition was calculated as the dependent variable. A 4 × 2 × 3 repeated measure ANOVA on the percentage of “synchronous” responses was conducted. The within-participant variables were Stimulus reliability (audio-visual intact, visual_intact/auditory_blurred, visual_blurred/auditory_intact, and audio-visual blurred), Audio-visual causality (high and low) and SOA (0, 200, and 400 ms). Greenhouse-Geisser correction was carried out for the results that did not meet the spherical hypothesis. Multiple comparisons were based on Tukey's T test.

### Results

The mean percentage of “synchronous” responses in each experimental condition is shown in Table 2. A 4 × 2 × 3 repeated-measures ANOVA revealed that the main effect of Stimulus reliability was significant [*F*_(3,87)_ = 6.51, *p* < 0.001, $\eta_{p}^{2}$ = 0.183], indicating that the percentage of synchronous responses in the audio-visual intact condition (53.5%) was significantly higher than that in the visual_intact/auditory_blurred condition (48.3%), *t* = 4.18, *p* < 0.001, *d* = 0.31 or in the audio-visual blurred condition (49.5%), *t* = 3.23, *p* = 0.009*, d* = 0.24. The main effect of SOA was also significant [*F*_(2,58)_ = 374.86, *p* < 0.001, $\eta_{p}^{2}$ = 0.928], indicating that the percentage of synchronous responses in the 0 ms SOA group (89.2%) was significantly higher than that in the 200 ms SOA group (51.0%), *t* = 13.5, *p* < 0.001, *d* = 0.87. Moreover, the percentage of “synchronous” responses in the 200 ms SOA group was significantly higher than that in the 400 ms SOA group (11.5%), *t* = 13.9, *p* < 0.001, *d* = 0.90. The main effect of Audio-visual causality was not significant [*F*_(1,29)_ = 0.22, *p* = 0.644, $\eta_{p}^{2}$ = 0.007].

**Table 2:** Percentage of “synchronous” responses under each experimental condition.

**Stimulus reliability**	**Audio-visual causality**	**Mean**	**SE**
Audio-visual intact	High	51.8	2.2
	Low	55.3	2.4
Visual_intact/auditory_blurred	High	48.9	2.4
	Low	47.7	2.1
Visual_blurred/auditory_intact	High	50.6	2.3
	Low	51.5	2.2
Audio-visual blurred	High	49.8	2.4
	Low	49.2	2.0

**Figure 4:**
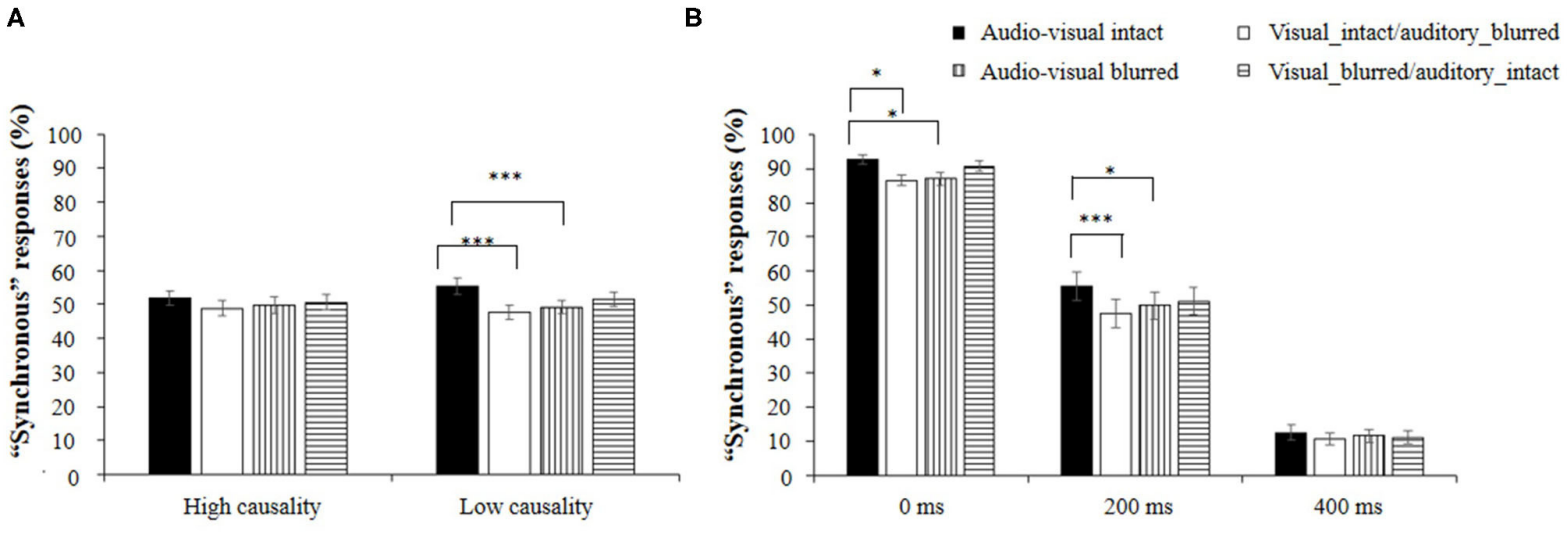
**(A)** Percentage of “synchronous” responses under different combined experimental conditions of Stimulate reliability and Audio-visual causality; **(B)** Percentage of “synchronous” responses under different combined experimental conditions of Stimulus reliability and SOA. *p < 0.05 and ***p < 0.001.

Most importantly, a significant interaction between Stimulus reliability and Audio-visual causality [*F*_(3,87)_ = 4.85, *p* = 0.004, $\eta_{p}^{2}$ = 0.143] was found. Further analyses showed that in the low causality condition, the percentage of “synchronous” responses in the audio-visual intact condition (55.3%) was significantly larger than that in both the visual_intact/auditory_blurred condition (47.7%), *t* = 5.32, *p* < 0.001, *d* = 0.56 and the audio-visual blurred condition (49.2%), *t* = 4.30, *p* < 0.001, *d* = 0.45. In contrast, no significant difference in the percentage of “synchronous” responses among different stimulus reliability conditions was found in the high causality condition (all *p* > 0.05), as shown in Figure 4A.

The interaction between Stimulus reliability and SOA was also significant [*F*_(6,174)_ = 3.02, *p* = 0.008, $\eta_{p}^{2}$ = 0.094]. Further analysis showed that when the SOA was 0 ms, the percentage of “synchronous” responses in the audio-visual intact condition (92.7%) was significantly higher than that in both the visual_intact/auditory_blurred condition (86.5%), *t* = 3.76, *p* = 0.012*, d* = 0.49 and the audio-visual blurred condition (87.0%), *t* = 3.45, *p* = 0.033*, d* = 0.44. When the SOA was 200 ms, the percentage of “synchronous” responses in the audio-visual intact condition (55.5%) was significantly higher than that in both the visual_intact/auditory_blurred condition (47.5%), *t* = 4.83, *p* < 0.001, *d* = 0.62 and the audio-visual blurred condition (49.8%), *t* = 3.45, *p* = 0.033*, d* = 0.44. In contrast, the percentage of “synchronous” responses did not differ significantly between each pair of stimulus reliability conditions when the SOA was 400 ms (see Figure 4B).

### Discussion

In the present study, stimulus reliability could significantly influence audio-visual synchrony perception, i.e., blurred stimuli are less likely to be perceived as synchronous than intact stimuli, which is contrary to our expectation. However, our results are consistent with the research of Shahin et al. (2017) and Shatzer et al. (2018), in which the authors blurred videos by using Gaussian filtering. They found that improving the time-frequency reliability of visual and auditory stimuli could promote audio-visual integration, thereby making synchrony perception more likely to occur.

For the percentage of “synchronous” responses, a significant interaction between stimulus reliability and audio-visual causality was found. In the low causality condition, for audio-visual intact stimuli, participants were more prone to make synchronous judgments than they were for both visual_intact/auditory_blurred and audio-visual blurred stimuli. However, this effect of stimulus reliability was not present in the high causality condition. Previous studies have shown that visual information conveyed by mouth movement is complex and might change with phoneme type (Cappelletta and Harte, 2012). Some phonemes are easier to recognize than others; for example, the mouth shape of /b/ is easier to recognize than that of /k/. That is, the visually obvious bilabial sounds used in the high causality condition could provide more visual information for participants to predict the auditory stimulus. In the high causality condition, reducing stimulus reliability did not affect synchronous judgment because there was enough information for participants to make judgments. However, in low causality, limited visual cues could be used to facilitate synchronous judgment.

The results of Experiment 2 were consistent with those of Experiment 1, although different measurements were adopted in these two experiments. In Experiment 1, participants' JNDs were smaller in the condition of high causality than they were in the condition of low causality, indicating that the participants were more sensitive to high causality stimuli than to low causality stimuli. Similarly, in Experiment 2, stimulus reliability could not influence synchrony judgment under the condition of high causality compared with that under the condition of low causality. These results indicated that enough visual cues were provided by high causality stimuli.

In addition, we also found that the interaction between stimulus reliability and AVOA was significant. When the SOA was 0 ms, participants were more likely to make synchronous judgments regarding audio-visual intact stimuli than for both visual_intact/auditory_blurred and audio-visual blurred stimuli. Similarly, when the SOA was 200 ms, participants were more tolerant of AVOA of audio-visual intact stimuli than that of both visual_intact/auditory_blurred and audio-visual blurred stimuli. Our results were consistent with those of Shatzer et al. (2018) and Shahin et al. (2017) when AVOA was smaller, i.e., when the auditory stimulus and visual stimulus were more synchronous, and stimulus reliability was higher, participants were more likely to make synchronous judgments.

## General Discussion

The aim of the present study is to investigate the effects of audio-visual causality and stimulus reliability on audio-visual synchrony perception. Audio-visual causality was manipulated by the audio-visual cues, i.e., visual stimuli predicted the onset of auditory stimuli. In Experiment 1, the JND of action stimuli was significantly smaller than that of speech stimuli in the high causality condition. In contrast, the JNDs between action and speech stimuli did not differ in the low causality condition. Similarly, the PSS of action stimuli was significantly different from that of speech stimuli in the high causality condition, whereas this effect disappeared in the low causality condition. In Experiment 2, stimulus reliability had a great impact on audio-visual synchrony perception in the low causality condition, i.e., the percentage of “synchronous” responses in the audio-visual intact condition was significantly larger than that in both the audio-visual blurred condition and the visual_intact/auditory_blurred condition. However, this effect of stimulus reliability disappeared in the high causality condition.

Our results show that the JND in action stimuli was smaller than that in speech stimuli under the high causality condition. Compared with non-speech stimuli, people were more tolerant of AVOA of speech stimuli, which was reflected in the wider temporal widow of integration (TWI) of speech stimuli (Dixon and Spitz, 1980; Stevenson and Wallace, 2013). In addition, even under the high causality condition, it was more difficult to see the movement of all the vocal organs, including the lips, teeth, throat, nose and other organs, in the video for speech stimuli than in the video for action stimuli; therefore, a larger JND was observed for speech stimuli than for action stimuli. In contrast, under low causality, the JNDs between action stimuli and speech stimuli were not significant. In addition, our results showed that participants were more sensitive to AVOA in the high causality condition than in the low causality condition for both action stimuli and speech stimuli, indicating that audio-visual causality modulates synchrony perception. Our results are in line with the findings of Eg and Behne (2015). In their study, the sensitivity to the AVOA of the action of playing chess was higher than that of speech stimuli, although no significant difference was found between the action of beating the drum and speech stimuli.

We found that participants were more tolerant of AVOA of speech stimuli, which might have been confounded by stimulus complexity. Vroomen and Stekelenburg (2011) found that the difference between speech stimuli and non-speech stimuli was not significant when the complexity of stimuli was manipulated. Therefore, in Experiment 2, the complexity of speech stimuli was controlled by manipulating the stimulus reliability. We found that stimulus reliability and audio-visual causality jointly affected the audio-visual integration of speech stimuli. Under the low causality condition, participants tended to make more synchronous judgments of the stimuli in the audio-visual intact condition than in both the visual_intact/auditory_blurred condition and the audio-visual blurred condition. In contrast, stimulus reliability did not affect audio-visual integration under the high causality condition. Our results indicated that the audio-visual causality might also play an important role in the synchrony perception, and the modulation of stimulus reliability on audio-visual synchrony perception was affected by the audio-visual causality. Under the high causality condition, visual stimuli provide ample information for auditory stimuli, while insufficient information is provided under the low causality condition. Therefore, participants are hard to make a judgment due to insufficient information under the condition of low causality, resulting in the tolerance to AVOA. By contrast, with abundant information, the decrease of stimulus reliability may not influence the synchrony perception under the condition of high causality.

Our results could be explained by the dynamic reweighting model (DRM) proposed by Bhat et al. (2015). They postulated that simple stimuli (such as pure tone) or unreliable auditory speech stimuli are more dependent on the lower auditory network, e.g., the primary auditory cortex and surrounding areas, considering that no complex speech information is available. With the increase in complexity and reliability of speech information, more information about speech and language is processed, and neural processing from low-level auditory networks is re-weighted to high-level auditory networks, e.g., the superior temporal sulcus, superior temporal cortex, and middle temporal gyrus. Through the process of re-weighting, the neural activity associated with the simple characteristics of sound decreases, and the neural activity associated with advanced pronunciation and vocabulary characteristics increases. During the process of re-weighting, the ability of the auditory system to discriminate time declines, and people are more tolerant of AVOA and more prone to synchronous judgment. Thus, we observed that the JND of speech stimuli was larger than that of action stimuli due to the complex process of re-weighting for speech stimuli. Besides, a previous study showed that the complexity of stimuli is reduced by decreasing stimulus reliability (Shatzer et al., 2018). The present study showed that the influence of stimulus reliability on synchrony perception is regulated by audio-visual causality. In the low causality condition, increasing the stimulus reliability, which could make a stimulus more complex, led to the re-weighting of audio-visual processing, which caused more synchronous judgment responses. In contrast, high stimulus reliability does not necessarily lead to high-level auditory network re-weighting due to the abundance of visual information in the high causality condition.

Our research also has some limitations. First, no difference was found between intact stimuli and visually blurred/auditory intact stimuli, which might be due to the visual stimuli being blurred with a Gaussian kernel. Pan and Bingham (2013) found that visual motion could be perceived even for blurred images. Thus, we found that the difference between intact stimuli and visually blurred stimuli disappeared, which could be attributed to the maintenance of visual information for motion perception. Second, in Experiment 2, we adopted response percentages as the dependent variable instead of evaluating the JND and PSS. Although the response percentage could be used to directly reflect audio-visual synchrony perception, comparison between the results from the two experiments is limited.

In summary, the present study supports the modulation effect of audio-visual causality and stimulus reliability on audio-visual synchrony perception. Moreover, our results support that there are co-effects of top-down and bottom-up factors on audio-visual synchrony perception. In the future, other audio-visual complex stimuli could be used to examine the impact of causality and other factors on the perception of simultaneity.

## Data Availability Statement

The raw data supporting the conclusions of this article will be made available by the authors, without undue reservation.

## Ethics Statement

The studies involving human participants were reviewed and approved by Ethics Committee of the Department of Psychology, Sun Yat-sen University. The patients/participants provided their written informed consent to participate in this study.

## Author Contributions

ZY, QD, and SL designed the research and wrote the manuscript. QD performed the research. SL, QD, and YY analyzed the data. All authors contributed to the article and approved the submitted version.

## Conflict of Interest

The authors declare that the research was conducted in the absence of any commercial or financial relationships that could be construed as a potential conflict of interest.
