# Supplementary material for: Audio-Visual Causality and Stimulus Reliability Affect Audio-Visual Synchrony Perception
Source: Front Psychol. 2021 Feb 18;12:629996. doi: 10.3389/fpsyg.2021.629996 (PMC7930005; doi:10.3389/fpsyg.2021.629996)
Supplement: Supplementary file 1 [file Table_1.DOCX]

Supplementary Material

# Supplementary Figures and Tables

**Supplementary Table 1**. Audiovisual clips under each experimental condition. Word frequency (Number per million words) is in parenthesis. The picture in the table is one frame selected from the audiovisual clips.

|  | High Causality | | Low Causality | |
| --- | --- | --- | --- | --- |
| Experiment 1  Action | 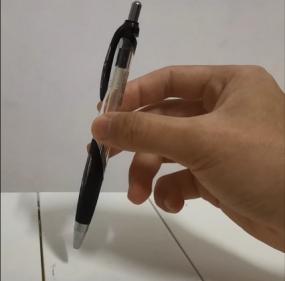  “Tap the table with a ballpoint pen” | | 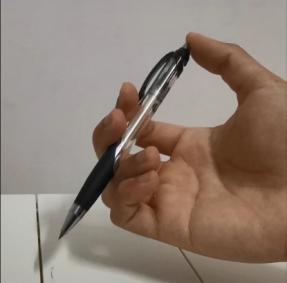  “Press the cap of the ballpoint pen” | |
|  | 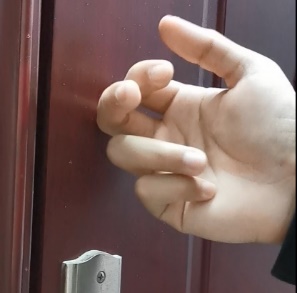  “Knock the door” | | 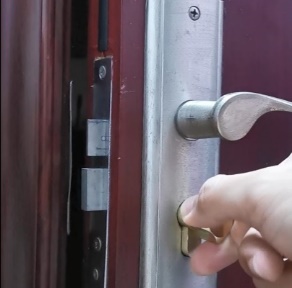  “Turn the security lock” | |
|  | 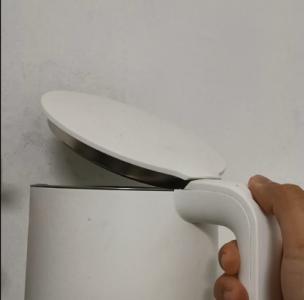  “Open and close the lid of the kettle” | | 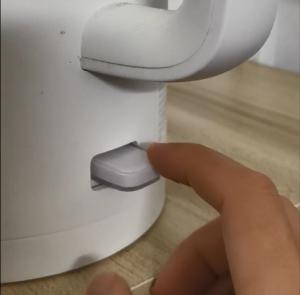  “Turn the kettle on and off” | |
| Speech | “拍卖品”(Lot) (0.34) | | “所在地”(Location) (2.17) | |
|  | “面包片”(Bread) (0.37) | | “淘汰赛”(Knockout) (0.9) | |
|  | “报名表”(Registration Form) (0.29) | | “甜丝丝”(Sweet) (0.07) | |
| Experiment 2 |  | |  | |
| Speech | “拍卖品”(Lot) (0.34) | | “所在地”(Location) (2.17) | |
|  | “面包片”(Bread) (0.37) | | “淘汰赛”(Knockout) (0.9) | |
|  | “报名表”(Registration Form) (0.29) | “甜丝丝”(Sweet) (0.07) | |  |
|  | “白茫茫”(Cloud-covered) (5.63) | “团体赛”(Team event) (0.05) | |  |
|  | “密麻麻”(Dense) (0.03) | “最低点”(Lowest point) (0.22) | |  |
|  | “排名表”(Ranking table) (0.02) | “点点头”(Nod head) (1.3) | |  |
